# Blood pressure reduction, persistence and costs in the evaluation of antihypertensive drug treatment – a review

**DOI:** 10.1186/1475-2840-8-18

**Published:** 2009-03-27

**Authors:** Peter Bramlage, Joerg Hasford

**Affiliations:** 1 Institute for Cardiovascular Pharmacology and Epidemiology, Mahlow, Germany; 2 Institut für Medizinische Informationsverarbeitung, Biometrie und Epidemiologie, University of München, Munich, Germany

## Abstract

**Background:**

Blood pressure lowering drugs are usually evaluated in short term trials determining the absolute blood pressure reduction during trough and the duration of the antihypertensive effect after single or multiple dosing. A lack of persistence with treatment has however been shown to be linked to a worse cardiovascular prognosis. This review explores the blood pressure reduction and persistence with treatment of antihypertensive drugs and the cost consequences of poor persistence with pharmaceutical interventions in arterial hypertension.

**Methods:**

We have searched the literature for data on blood pressure lowering effects of different antihypertensive drug classes and agents, on persistence with treatment, and on related costs. Persistence was measured as patients' medication possession rate. Results are presented in the form of a systematic review.

**Results:**

Angiotensin II receptor blocker (ARBs) have a competitive blood pressure lowering efficacy compared with ACE-inhibitors (ACEi) and calcium channel blockers (CCBs), beta-blockers (BBs) and diuretics. 8 studies describing the persistence with treatment were identified. Patients were more persistent on ARBs than on ACEi and CCBs, BBs and diuretics. Thus the product of blood pressure lowering and persistence was higher on ARBs than on any other drug class. Although the price per tablet of more recently developed drugs (ACEi, ARBs) is higher than that of older ones (diuretics and BBs), the newer drugs result in a more favourable cost to effect ratio when direct drug costs and indirect costs are also considered.

**Conclusion:**

To evaluate drugs for the treatment of hypertension several key variables including the blood pressure lowering effect, side effects, compliance/persistence with treatment, as well as drug costs and direct and indirect costs of medical care have to be considered. ARBs, while nominally more expensive when drug costs are considered only, provide substantial cost savings and may prevent cardiovascular morbidity and mortality based on the more complete antihypertensive coverage. This makes ARBs an attractive choice for long term treatment of hypertension.

## Background

Blood pressure lowering drugs are approved based on short term trials determining the absolute blood pressure reduction during trough and the duration of the antihypertensive effect after single or multiple dosing. The absolute amount of blood pressure reduction in mmHg over the short term can however not be extrapolated to the degree of protection against hypertensive end organ damage because low patient's compliance and poor persistence with treatment may lead to early discontinuation of treatment in clinical practice [1-3].

To be effective treatment must continue, sometimes for a patient's life, despite an absence of symptoms or any perceived benefit to the patient [4-6]. Unfortunately, lack of symptoms in hypertension together with a low tolerability of some antihypertensive drugs are some of the most common reasons for patients discontinuing treatment or not taking the medication at the prescribed dose and at the required intervals. A poor compliance/persistence in turn, leads to an increase in the use of healthcare resources and an increase in overall expenditure [7]. Thus, poor persistence has been recognised as a serious problem with significant economic consequences. Although studies have investigated the extent of the economic effect of non-compliance, such studies evaluated different aspects of this effect and are not able to give a complete picture.

Therefore this review explores the cost consequences of poor persistence with pharmaceutical interventions in arterial hypertension. The aim is 1) evaluating the antihypertensive effects of drugs, 2) reviewing persistence with different pharmacotherapies and 3) exploring the related expenditure, such as drug costs, overall healthcare expenditure and productivity costs, and investigating the effect it has on the cost-effectiveness of pharmaceutical interventions for hypertension.

## Materials and methods

### Definitions

In this review, the definitions of the International Society for Pharmacoeconomics and Outcomes Research (ISPOR) were used, which define compliance as taking medication as prescribed, on time and at the correct dose, and persistence as the continuing use in time of the prescribed therapy [8]. Defined daily doses (DDDs) based on the assumed average maintenance dose per day were used to compare costs.

### Searches

We have identified studies describing compliance/persistence with treatment using different antihypertensive classes and their related costs. The following search term was entered into pubmed: "hypertens* AND (complia* OR adhere* OR persiste*) AND (cost* OR econo*)" with the limits: "Publication Date from 1995/01/01, Humans, English." A manual search of the reference lists from retrieved publications was also performed to identify further relevant studies.

### Selection criteria

Studies were regarded relevant if they were in English language, involved human studies published before November 2008, involved patients with hypertension, examined compliance (adherence) and/or persistence to pharmaceutical interventions (even if the primary objective was not to measure compliance/persistence), provided an economic evaluation or cost analysis and quantified the cost consequences of compliance/persistence. Studies published before 1995 were excluded as results from those earlier studies could not be compared with those from more recent studies because of changes in treatment patterns, study methodology and the price of healthcare resources, including drug prices. Studies were also excluded from analysis if the economic consequence of compliance/persistence was not quantified.

### Data extraction

Qualitative data extracted from the studies included the country where the study was performed, the number of patients, the database used, the type of study (retrospective, prospective, model or based on assumptions), the duration of follow-up, definitions used for persistence, compliance, switching and discontinuation as well as the type of patients with mean age, gender and drugs at the onset of the study (see Table 1a &1b).

**Table 1:** Overview of studies on compliance / persistence with treatment

**a – Studies on compliance/persistence with treatment (Part 1)**					
**References**	**Database**	**Study design**	**Definitions**	**Patients**	**Baseline**

Bloom 1998 [15] USA 21,723 pts.	Bloom cohort study, longitudinal database with pharmacy administrative claims [15]	Retrospective, longitudinal cohort study 1 year follow-up	Persistence: at least 3 refills over 1 year Switch: no initial AH drug but other AH Discontinued: ≤ 2 refills	Patients with initiation on antihypertensive monotherapy, and no treatment within the 12 month before	Mean age: 56 y Male: 44.1% Initial drug class: ARBs: 2.6% ACEi: 26.9% CCBs: 23.4% BBs: 23.0% Diuretics: 24.1%
Rizzo 1996 [27] USA Pts. not available	National Medical Expenditure Survey 1987	Retrospective, longitudinal cohort study 1 year follow-up	not available	Patients with chronic illnesses including hypertension	Age range 18–64 y
Rizzo 1997 [25] USA 7,211 pts.	Pennsylvania Medicaid Management Information System	Retrospective, longitudinal cohort study 1 year follow-up	Compliance: estimated	Patients with a treatment for hypertension (monotherapy)	Mean age: 59.4 y Male: 29% Initial drug class: ACEi: 32% CCBs: 49% BBs: 11% Diuretics: 8%
Hughes 1998 [26] UK 7,741 pts.	MEDIPLUS database	Retrospective, longitudinal cohort study 26 months follow-up	Persistence: patients continuing therapy at 6 months	Patients with new antihypertensive treatment	Not available
Conlin 2001 [18] USA 15,175 pts.	Bloom cohort study, longitudinal database with pharmacy administrative claims [15]	Retrospective, longitudinal cohort study 1 + 3 year follow-up	Persistence: at least 3 refills over 1 year Switch: no initial AH drug but other AH Discontinued: ≤ 2 refills	Patients with initiation on antihypertensive monotherapy, and no treatment within the 12 month before	Mean age: 56 y Male: 45% Initial drug class: ARBs: 3.0% ACEi: 29.2% CCBs: 25.6% BBs: 25.1% Diuretics: 17.1%
Hasford 2002 [36] Germany, France, UK 2,416 pts.	IMS MediPlus	Retrospective, longitudinal cohort study 1 year follow-up	Persistence: 4 refills over 1 year Switch: no initial AH drug but other AH Discontinued: stopped refill for at least 30 days after medication ran out	Patients with initiation on antihypertensive monotherapy, no previous treatment	Mean age: 61 Male: ~50% Initial drug class: ARBs: 31.2% ACEi: 13.8% CCBs: 19.3% BBs: 18.3% Diuretics: 17.5%
Degli Esposti 2001 Italy 4,614 pts.	Local Health Unit of Ravenna	Retrospective, longitudinal cohort study		Patients with a first prescription for amlodipine, atenolol fosinopril, indapamide or losartan	
Degli Esposti 2002 [22] Italy 7,312 pts.	Local Health Unit of Ravenna	Retrospective, longitudinal cohort study 3 year follow-up	Continuers (persistence): at least 2 prescriptions per year over 3 years Discontinuers: at least 2 prescriptions per year for the first year only or and the second year but not in the third year	Patients with new antihypertensive drug treatment	Mean age: ~63 y Male: 43.1 Initial drug class: ARBs: 2.7% ACEi: 33.1% CCBs: 25.7% BBs: 15.9% Diuretics: 22.6%

**b – Studies on compliance/persistence with treatment (Part 2)**					

**References**	**Database**	**Study design**	**Definitions**	**Patients**	**Baseline**

Degli Esposti 2004 [23] Italy 14,062 pts.	Local Health Unit of Ravenna	Prospective, longitudinal cohort study 1 year follow-up	Persistence: therapy duration of > 273 days.	Patients with new antihypertensive drug treatment	Mean age: 56.9 y Male: 43.4% Initial drug class: ARBs: 6.9% ACEi: 28.0% CCBs: 23.8% BBs: 17.6% Diuretics: 23.8%
Erkens 2005 [37] Netherlands 2,243 pts.	PHARMO database including pharmacy records and hospitalisation	Retrospective, longitudinal cohort study 1 year follow-up	Persistence: drugs for at least 270 d and 3 month after the 1 year follow-up Switch: no initial AH drug but other AH Discontinued: stopped refill for at least 30 days after medication ran out	Patients with initiation on antihypertensive monotherapy, no previous treatment	Mean age: most pts. (42.6%) 40–59 y Male: 43.1% Initial drug class: ARBs: 19.9% ACEi: 18.4% CCBs: 20.3% BBs: 21.0% Diuretics: 20.4%
Sokol 2005 [24] USA 7,981 pts.	Participants of a medical and drug benefit plan sponsored by a large manufacturing employer	Retrospective, longitudinal cohort study 1 year follow-up	Adherence was defined as the percentage of days during the analysis period that patients had a supply of *1 or more *maintenance medications for the condition (based on "days' supply" data in patients' prescription claim records).	Patients being on the respective treatment for at least 12 months.	Mean age: 54.2 y Male: 53.3%
Veronesi 2007 [19] Italy 347 pts.	Outpatients of a hypertension clinic	Prospective, longitudinal, single-blind, cohort study 2 year follow-up	Persistence: continued use of the initial AH over time	Patients with initiation on antihypertensive monotherapy, no antihypertensive treatment in the last 6 month	Mean age: 59.4 y Male: 59.4% Initial drug class: ARBs: 15.2% ACEi: 17.5% CCBs: 18.1% BBs: 17.5% Diuretics: 18.1%
Hasford 2007 [17] Germany 13,763 pts.	IMS disease analyse database	Retrospective, longitudinal cohort study 3 year follow-up	Persistence: continued treatment over time Discontinuation: no repeat AH drug prescription for more than 6 month	Patients with initiation on antihypertensive monotherapy or a specified combination, no antihypertensive treatment in the last 6 month	Median age: 65 y Male: 44.2% Initial drug class: ARBs: 5.4% ACEi: 21.0% CCBs: 11.8% BBs: 37.3% Diuretics: 14.5%
Patel 2007 [16] USA 242,882 pts.	Administrative pharmacy claims from MedImpact	Retrospective, longitudinal cohort study 1 year follow-up	Persistence: continued treatment over time Discontinuation: no refill for 60 days after running out of pills	Patients with initiation on antihypertensive monotherapy	Median age: 54.5 y Male: 43.1% Initial drug class: ARBs: 4.2% ACEi: 32.4% CCBs: 14.9% BBs: 34.1% Diuretics: 14.4%

ARBs, Angiotensin receptor blockers; ACEi, Angiotensin converting enzyme inhibitor; CCBs, calcium channel blockers; BBs, Beta-blockers; y, years

## Studies on the antihypertensive effect of drugs

As the number of studies well exceeded the handling limit and an excellent meta-analysis on the blood pressure lowering effect of antihypertensive drugs was available, we referred to the work of Law and colleagues [9], who analyzed the data from 354 randomized, placebo controlled studies. 39,879 patients in all trials received active treatment and 15,817 received matching placebo. Active treatment options investigated were angiotensin II receptor blockers (ARBs), ACE-inhibitors (ACEi), calcium channel blockers (CCBs), beta-blockers (BBs) and diuretics. The pre-treatment blood pressure (mmHg) was 154 (90%CI 139 to 170)/97 (87 to 106) in both the treatment and placebo group. The median duration of the trials was 4 weeks (90%CI 2–12).

Results are summarized in Table 2, illustrating a strong reduction of systolic blood pressure with ARBs (10.3 [95%CI 9.9 to 10.8]) and a particularly strong reduction of diastolic blood pressure with BBs (6.7 [95%CI 6.2 to 7.1]). The average fall in blood pressure across the five categories was 9.1 mmHg systolic and 5.5 mmHg diastolic. Within each class there was no evidence that any specific drug was substantially better than the others. ARBs on average provided a better blood pressure reduction than ACEi (net difference 1.8/1.0 mmHg), which could translate into a reduction in morbidity and mortality [10].

**Table 2:** Average reduction in blood pressure over 24 hours (treated minus placebo) according to category of drug and dose [9]

**Drugs**	**Systolic/Diastolic**	**Fall in blood pressure (mmHg (95%CI))**
Thiazides	Systolic	8.8 (8.3 to 9.4)
	Diastolic	4.4 (4.0 to 4.8)
Beta-blockers	Systolic	9.2 (8.6 to 9.9)
	Diastolic	6.7 (6.2 to 7.1)
ACE inhibitors	Systolic	8.5 (7.9 to 9.0)
	Diastolic	4.7 (4.4 to 5.0)
Angiotensin II receptor antagonists	Systolic	10.3 (9.9 to 10.8)
	Diastolic	5.7 (5.4 to 9.0)
Calcium channel blockers	Systolic	8.8 (8.3 to 9.2)
	Diastolic	5.9 (5.6 to 6.2)

In each category, the fall in blood pressure was standardised to the average starting blood pressure across all trials of 154 mmHg systolic and 97 mmHg diastolic; the estimates are the average over 24 hours from combining separate peak and trough estimates.

Law et al. also investigated the reported adverse events from these studies in their meta-analysis (Table 3). Symptoms attributable to thiazides, BBs, and CCBs were strongly dose related; symptoms caused by ACEi (mainly cough) were not dose related. ARBs caused no excess of symptoms.

**Table 3:** Adverse effects of drugs: percentage of people with one or more symptoms attributable to treatment*, according to category of drug and dose, in randomised trials [9,20]

		**Percent (95%CI) with symptoms (treated minus placebo)**^†^		
**Drug class**	**No. of trials**	**1/2 standard dose**	**Standard dose**	**Twice standard dose**
Thiazides	59	2.0 [-2.2 to 6.3]	9.9 [6.6 to 13.2]	17.8 [11.5 to 24.2]
BBs	62	5.5 [0.3 to 10.7]	7.5 [4.0 to 10.9]	9.4 [3.6 to 15.2]
ACEi	96	3.9 [-3.7 to 11.6]	3.9 [-0.5 to 8.3]	3.9 [-0.2 to 8.0]
ARBs	44	-1.8 [-10.2 to 6.5]	0 [-5.4 to 5.4]	1.9 [-5.6 to 9.3]
CCBs	96	1.6 [-3.5 to 6.7]	8.3 [4.8 to 11.8]	14.9 [9.8 to 20.1]

ACE = angiotensin converting enzyme. *Calculated as difference between treated and placebo groups in proportion of participants who developed one or more symptoms, excluding headaches, which were significantly less common in people receiving treatment. ^†^Commonest symptoms: thiazides–dizziness, impotence, nausea, muscle cramp; Beta-blockers–cold extremities, fatigue, nausea; ACE inhibitors–cough; calcium channel blockers–flushing, ankle oedema, dizziness.

Real-life data support the key findings of this meta-analysis. The UK THIN general practice database with 16,866 records of patients receiving ACEi (14,651 pts) or ARBs (2,215 pts) was analysed for example for the degree of blood pressure reduction [11-14]. At one year, mean systolic blood pressure reductions for patients receiving ARBs reached 13.2 mmHg compared to 11.1 mmHg for patients receiving ACEi (diastolic 7.8 vs. 6.7 mmHg). Similar results were also observed after total treatment duration of 2 years. The comparisons were significant (p < 0.001) in a linear mixed multivariate model adjusting for a number of confounding factors.

## Studies on persistence with treatment

Table 4 displays the persistence with initial treatment in 8 studies ranging from 1 to 4 years of observation. In most cases the persistence with ARBs was highest, with 12 month values of between 42 and 64%.

**Table 4:** Persistence with initial treatment in different studies

	**Duration**	**ARBs**	**ACEi**	**CCBs**	**BBs**	**Diuretics**
**Bloom **[15]	12	64%	58%***	50%	43%	38%
**Conlin **[18]	48	50.9%	46.5%	40.7%**	34.7%**	16.4%**
**Hasford **[36]	12	51.3%	42.0%	43.6%	49.7%	34.4%
**Degli-Esposti **[23]	12	41.7%	32.2%	26.7%	36.9%	25.9%
**Erkens **[37]	12	62.0%	59.7%	34.7%	35.0%	33.0%
**Veronesi **[19]	24	68.5%	64.5%	51.6%**	44.8%**	34.4%*
**Hasford **[17]	12	26.4%	28.2%	25.9%	25.8%	21.9%
**Patel **[16]	12	51.9%	48.0%	38.3%	40.3%	29.9%

Duration is given in month. *p < 0.01; **p < 0.05; *** p < 0.007 vs. ACEi

The study by *Bloom et al*. [15] reported a lower persistence with ACEi (OR 0.81; 95%CI 0.68–0.97), CCBs (OR 0.62 [95%CI 0.51–0.74]), BBs (OR 0.56 [95%CI 0.47–0.68]) and thiazides (OR 0.36 [95%CI 0.30–0.43]) than with ARBs (reference) over a 1 year period. Higher age (≥ 65 years) and once daily dosing were also identified to increase persistence vs. young age or multiple dosing. *Conlin et al*. reported the 4 year follow-up of this cohort, which essentially resulted in compatible results, although persistence rates further dropped over the 4 year follow-up. At 4 years ARBs had the highest persistence with 50.9% of patients still being on monotherapy with a stepwise decline for the other drug classes. A further study by Veronesi reported that the persistence with ARBs was generally high (68.5%) while ACEi (OR 0.94; 95%CI: 0.79–0.99), CCBs (OR 0.76; 95%CI: 0.54–0.85), BBs (OR 0.67; 95%CI 0.57–0.79) and thiazide diuretics (OR 0.56; 95%CI 0.38–0.84) had a lower persistence.

**Figure 1:**
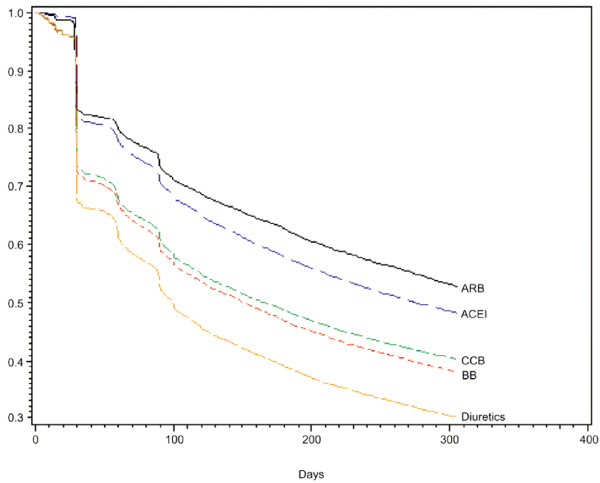
**Time to therapy discontinuation of antihypertensive monotherapy **[16]. This depicts the proportion of study patients by antihypertensive medication class who remained persistent with index therapy (y axis) during the year subsequent to the index study claim (days subsequent to index date depicted on x axis). ARB patients were most likely to remain on therapy, closely followed by ACEi patients. Diuretic patients were least likely to remain on the index monotherapy regimen.

A study by Patel et al. reported, in addition to persistence rates, a survivor function estimate (time to therapy discontinuation) which is depicted in Figure 1[16]. Most patients who discontinued therapy did so within the first 30 days of starting therapy. The differences between index drug classes observed at 12 months post-index date were largely evident at 1 month post-index date. Compared with patients receiving diuretics, those receiving ARBs (HR 0.593; p < 0.0001), ACEi (HR 0.640; p < 0.0001), CCBs (HR 0.859; p < 0.0001), and BBs (HR 0.819; p < 0.0001) were all less likely to discontinue therapy.

One study however reported generally lower persistence rates for all drug classes [17]. The persistence with ARBs in this study was 26.4%, thus being lower than those of ACEi with 28.2%.

Overall, patients on ARBs were more compliant than patients on ACEi and CCBs, BBs and diuretics.

## Blood pressure × persistence product

**Figure 2:**
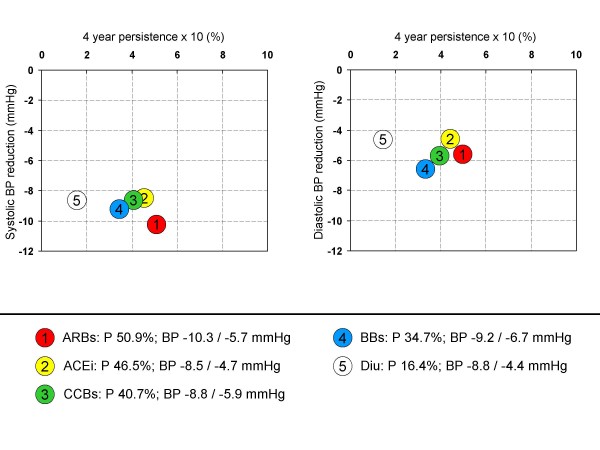
**Blood pressure reduction vs. 4 year persistence with treatment**. Mean systolic blood pressure reduction was taken from Law [9], 4 year persistence with monotherapy was taken from Conlin [18].

The blood pressure × persistence product could provide a more meaningful insight into the true antihypertensive effectiveness of antihypertensive drugs. Two approaches were chosen to illustrate differences in the blood pressure × persistence product:

1.) Placebo corrected blood pressure reductions reported in the meta-analysis of Law et al. [9] were multiplied with the persistence pattern at 1 year reported by Conlin et al. [18] (Figure 2). The results show that ARBs, although having only slight advantages in systolic (but not diastolic) blood pressure reduction, resulted in the longest persistence with treatment over a period of 4 years. Persistence and blood pressure reduction with diuretics were on the other hand low as compared to the other drug classes. The strengths of this approach are that placebo corrected blood pressure reductions have been used from a large number of placebo controlled trials. Blood pressure reductions were based on the usual maintenance dose of drugs. The median treatment duration of all trials in this analysis was however only 4 weeks which might result in an underestimation of true antihypertensive efficacy especially for drugs with a slow onset of action. Further studies were included from a large time range, thus giving rise to the notion that effects and treatment schemes might be not comparable to the situation today.

**Figure 3:**
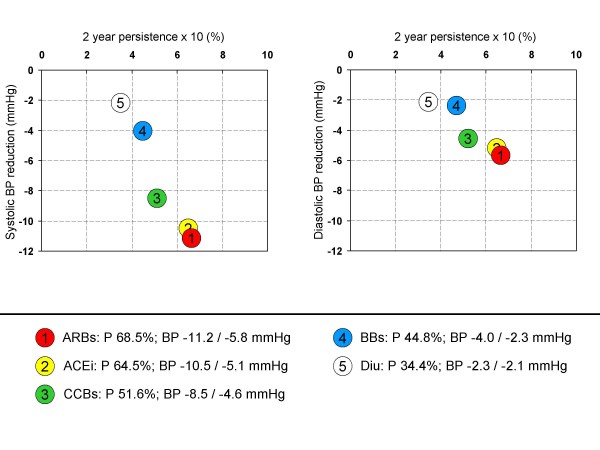
**Blood pressure reduction vs. 2 year persistence with treatment**. Mean blood pressure reduction and persistence with treatment over 2 years was taken from Veronesi [19].

2.) The study by Veronesi et al. [19] not only provided persistence with treatment but also the blood pressure lowering effect over a time frame of 2 years (Figure 3) from the very same patients. For this reason many of the limitations of the first approach do not apply. Compared to the aforementioned version differences in the blood pressure lowering effect between drug classes are substantial (within a range of -2.3 to -11.2 mmHg systolic and -2.1 to -5.8 diastolic). Patients treated with ARBs had the most effective blood pressure lowering (-11.2/-5.8 mmHg), followed by ACE inhibitors (-10.5/-5.1 mmHg). Both were significantly more effective than BB and diuretics (p < 0.05) but not vs. each other. The variability in persistence over 2 years is similar to the analysis above so that larger differences between drug classes are evident. However, the study did not record the dose used to achieve the antihypertensive effect, which may confound the results towards drugs used in higher doses. On the other hand drugs with a good tolerability in high doses (as has been reported for ACEi and ARBs by Law [20]) might result in greater blood pressure reduction achievable in line with a higher persistence.

## Costs and cost-effectiveness

14 studies were identified that analysed the effect of compliance and/or persistence on the cost or cost-effectiveness of a treatment for hypertension.

### Drug costs

Three studies investigated the drug costs with different compliance and persistence in antihypertensive drug treatment [21-23]. The early study by Degli-Eposti reported mean drug costs to be highest for patients adding another drug to their therapeutic regimen (combination therapy, 274.69 €) and lowest for occasional users (32.80 €) [21]. Patients who stayed on therapy were less costly (121.51 €) than those switching to another treatment (182.25 €).

In the 2004 study by Degli-Esposti [23], average drug costs were found to be lowest for patients on diuretics and those who discontinued therapy. Drug costs were highest for patients being treated and being persistent with ARB therapy (326.16 €), closely followed by patients persistent with CCB treatment (234.63 €) and patients switching between classes (up to 268.07 €). Details can be found in Table 5.

**Table 5:** Annual average drug costs per patient for different antihypertensives according to the pattern of persistence [23]

**Antihypertensive**	**Continuers** **[95%CI]**	**Switchers** **[95%CI]**	**Discontinuers** **95%CI]**	**Total** **[95%CI]**
Diuretics	€ 65.09 [58.67–71.52]	€ 153.10 [137.59–168.62]	€ 8.17 [7.60–8.75]	€ 33.45 [30.97–35.93]
Beta-blockers	€ 109.29 [102.46–116.12]	€ 158.73 [139.61–177.84]	€ 22.52 [24.24-23.79]	€ 63.40 [59.94–66.86]
Calcium-channel blockers	€ 234.63 [224.78–244.47]	€ 199.62 [183.45–215.78]	€ 38.24 [36.78–39.70]	€ 104.43 [100.07–108.79]
ACE inhibitors	€ 196.28 [189.69–202.86]	€ 237.53 [222.28–252.79]	€ 34.76 [33.53–35.99]	€ 108.25 [104.43–112.09]
Angiotensin II antagonists	€ 326.16 [313.05–339.27]	€ 268.07 [241.55–294.39]	€ 67.10 [62.89–71.31]	€ 201.53 [191.24–211.81]

**Total**	**€ 171.73** **[167.43–176.04]**	**€ 205.10** **[196.85–213.34]**	**€ 28.29** **[27.62–28.97]**	**€ 88.09** **[86.10–90.08]**

CI, confidence interval

### Direct costs

**Figure 4:**
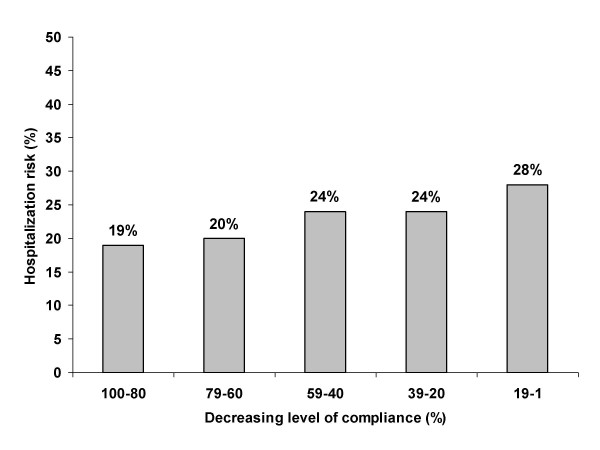
**Risk of hospitalisation (%) in relation to the level of compliance for hypertension **[24]. Level of compliance grouped into quintiles vs. risk of hospitalization. Patients with a high degree of compliance (of between 80 and 100%) have a 19% risk of hospitalization as compared to 28% in patients with a compliance between 1 and 19%.

**Figure 5:**
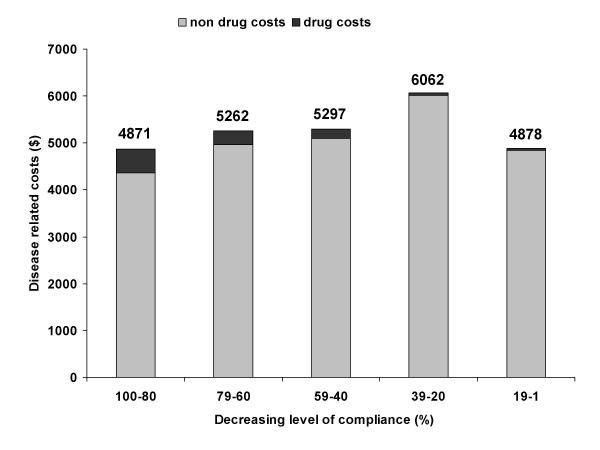
**Disease-related healthcare costs in relation to the level of compliance for hypertension **[24]. Costs are in $.

When other, non-drug direct costs are taken into account, the results are different. The impact of medication adherence on healthcare utilization and cost for 4 chronic conditions including hypertension was investigated in a retrospective cohort study in patients who were enrolled in a medical and prescription benefits plan [24]. Patients were identified for disease-specific analyses based on claims for outpatient, emergency room, or inpatient services during the first 12 months of the study. Using an integrated analysis of administrative claims data, medical and drug utilization were measured during the 12-month period after patient identification. Medication persistence was defined by days' supply of maintenance medications for each condition. All-cause costs were defined as any healthcare costs over a 1-year period. For these, 80–100% persistence with treatment for hypertension was associated with significantly lower non-drug medical costs than for levels below 80% (6570$ vs. 7658–10,286$; p < 0.05 for high vs. lower levels of compliance) (Table 6). Therefore higher levels of persistence with treatment were associated with lower overall healthcare costs, despite high drug costs. The decrease in healthcare costs with increasing persistence was attributed mainly to a decrease in the risk of hospitalisation (Figure 4). Similar associations were seen for disease related costs (Figure 5). Disease-related costs were defined as those associated with the disease only. In hypertension, a higher level of persistence was associated with lower disease-related costs. Results were however not statistically significant.

**Table 6:** Disease-Related Healthcare Costs and Hospitalization Risk at Varying Levels of Medication Adherence [24]

**Adherence Level**	**N**	**Medical Costs ($)**	**Drug Costs ($)**	**Total Costs ($)**	**Hospitalization Risk (%)**
1–19	350	4847	31	4878	28
20–39	344	5973	89	6062	24
40–59	562	5113	184	5297	24
60–79	921	4977	285	5262	20
80–100	5804	4383	489	4871	19

		F = 46.44**	F = 171.98**		X2 (31 df) = 1256.3**
		**Adj. r^2 ^= 0.13**	**Adj. r^2 ^= 0.37**		

CI, confidence interval; *Indicates that the outcome is significantly higher than the outcome for the 80–100% adherence group (P < 0.05). Differences were tested for medical cost and hospitalization risk; ** p < 0.0001

In a retrospective study of hypertensive patients [25], the highest direct costs were produced by patients changing their medication by switching or adding another antihypertensive drug (2142$), followed by non-persistent patients (735$, p = 0.05 vs. first group) and noncompliant patients (694$, p = 0.01 vs. first group). The lowest costs were seen with persistent patients (341$, p = 0.01 vs. first group). Similar relations although on a higher total level were observed for patients with co-morbid renal failure, acute myocardial infarction, diabetes, congestive heart failure and angina. Patients with renal failure produced the highest cost with 3936$ in patients changing their medication and lowest costs in persistent patients (2135$). However, the compliance/persistence data were based on self-report, making them prone to various kinds of bias.

In a UK study based on the MEDIPLUS data set, patients switching medication were again found to produce the highest drug costs (218£ vs. 192£ for continuers). Hospital costs were higher in patients either switching or discontinuing therapy (70£) as compared to persistent patients (46£). Total costs for patients were lower for continuers (280£) than for patients switching medication (336£) [26].

### Indirect costs

Only one study investigated both direct and indirect costs [27]. Besides drug costs, this study examined indirect costs in terms of days missed from work. The aim was to calculate the overall cost effects of employer-provided drug coverage and of an increase in compliance to 100%. Over a 1-year period with average co-payments of 63% in hypertension, employers acquired mean 22.28$ (corresponding to about 17.58 € as of December 2008) extra drug costs per employee.

Increased compliance resulted in 3.48–16.06 saved work days per employee. Assuming an average wage of 9.32$ per hour and fringe benefits of 25%, the benefit from avoiding missed work days was greater than the extra drug costs paid by the employers, resulting in a significant yearly net benefit to the employers (286 [high 366, low 205]$ per employee; Table 7). Assuming that compliance can be increased to 100%, the yearly saving in indirect costs would amount to 191$ per employee. However, as this assumption is not realistic, these savings can only be interpreted as upper limits of the potential savings.

**Table 7:** Benefits to the employer of employer-provided drug coverage and increasing compliance to 100% in hypertension treatment [27]

**Compliance level**	**Treatment effect (days saved)**	**Employer costs**	**Employer savings**	**Net benefit**
Average compliance (37% drug coverage)				
High	4.35	39 $	405 $	366 $
Base	3.48		325 $	286 $
Low	2.62		244 $	205 $

Additional benefit if compliance increased to 100%				
High	2.55	22 $	238 $	216 $
Base	2.05		191 $	169 $
Low	1.54		143 $	121 $

### Factors influencing cost effectiveness

**Figure 6:**
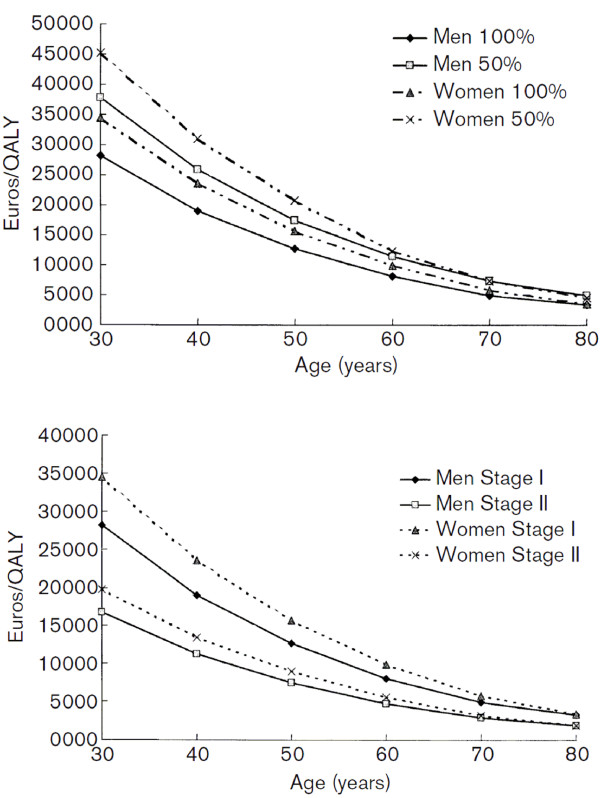
**Cost-effectiveness ratios of treatment of hypertension, by hypertension stage, sex and age. QALY, quality adjusted life year **[28]

Mar and colleagues evaluated the different parameters influencing the cost-effectiveness of antihypertensive drugs in patients with stage I and II arterial hypertension [28]. Direct non-medical costs and indirect costs were incorporated into some of the scenarios in addition to direct medical costs. The cost of an additional Quality Adjusted Life Year (QALY) varied substantially from 34,516€ in 30-year-old women to 3,307€ in 80 year old men. Treatment of arterial hypertension stage II showed lower ratios (19,798€/QALY in 30-year-old women and 1,918€/QALYin 80-year-old persons. The cost-effectiveness ratio decreased with increasing age and was less in men than in women (Figure 6). The inclusion of travel and productivity costs increased the cost-effectiveness ratio by 30% in women and by 35% in men. Assuming a linear relationship between compliance and efficacy, a decrease in compliance of 50% resulted in an increase in the incremental cost effectiveness ratio (ICER) of 30–50%. Cost-effectiveness ratios for arterial hypertension stage I vary from 645€/QALY in 80-year-old men for diuretics to 47 325€/QALY in 30-year old women for ACEi's. This increase was greater in older patients and was greater in men than women.

## Discussion

The mere consideration of the blood pressure lowering effect for the evaluation of antihypertensive drugs falls short if patients' compliance and persistence with treatment are not considered. This is evident from studies in which it was shown that control rates in hypertension are low [29-31]. On the basis of population studies across the world hypertension control rates have been reported as low as 53.1% for the US, 41.0% for Canada, 33.6% for Germany and 29.2% for the UK [32], which illustrate that the high control rates reported in randomized controlled trials do not always translate into clinical practice [33,34]. It is therefore particularly dangerous to utilize the blood pressure reduction from short term randomized trials to extrapolate to costs of treatment. Current attempts to limit the access to newer antihypertensive drug classes [35] have to fail in reducing the overall cost impact of hypertension, if persistence with treatment as well as non-drug costs of non-persistent patients are not taken into account.

Since there are no studies specifically addressing all aspects of antihypertensive treatment, it was therefore our aim to provide a comprehensive overview of the available data. Several key findings deserve mentioning:

• A comprehensive meta-analysis has shown differences in the antihypertensive efficacy between antihypertensive drug classes, favouring ARBs over other drug classes (ARBs > BBs > CCBs = thiazides > ACEi for systolic blood pressure) [9];

• Persistence with treatment is highest with ARBs (ARBs > ACEi > CCBs > BBs > diuretics) [15,16,18,19,23,36,37];

• Drug costs are however higher with ARBs than with any other drug class [21-23];

• A high persistence with drug treatment as can be observed with more recent antihypertensive drugs (ARBs, ACEi and CCBs) results in an over-compensation of direct drug costs, leading to a substantial decrease in direct and indirect costs, and may also translate into a reduction of morbidity and mortality [24-27,38].

There are however some open questions for which there is a weaker evidence base.

### Comparison of ARBs and ACEi after ONTARGET

After publication of the ONTARGET results [39,40] the discussion of whether ARBs or ACEi are superior antihypertensive drugs was halted for some, since both treatment regimens proved to be equally effective on the long term to reduce cardiovascular events in high risk patients with or without diabetes. In terms of blood pressure reduction the trial did not however provide further evidence since blood pressure at baseline was low already (blood pressure at baseline 142/82 mmHg) and only a subset of patients was hypertensive per common definitions (69%). Blood pressure was reduced by only 6.4/4.3 mmHg in the ramipril group and only 7.4/5.0 mmHg in the telmisartan group (difference of 0.9/0.6 mmHg in favour of telmisartan). The trial may however provide evidence that ARBs may not provide additional benefit over ACEi in case the blood pressure is already low in the above mentioned patient population.

### Differences among antihypertensive drugs within a class?

In their meta-analysis of 354 placebo controlled trials, Law et al. found, although there were nominal differences, no significant evidence for differences in the antihypertensive effect of different drugs within one class [9,20]: "*Within each category there was no evidence that any specific drug was materially better than the others*." Two aspects have to be considered when comparing different drugs within a class. 1) Defined daily doses (DDD) are not equally efficient doses. They were defined for statistical purposes on drug utilization only and represent the *"assumed average maintenance dose per day for a drug used for its main indication in adults." *This was illustrated in a study by Dominiak and colleagues who were able to demonstrate that in many cases 2 DDDs have to be prescribed to be equally effective to drugs prescribed at the usual DDD (Table 8) [41,42]. Comparisons based on these DDDs for ARBs have shown that the ARB irbesartan at 150 mg for example is more effective than losartan 50 mg [43,44] and valsartan 80 mg [45]. 2) As ARBs can be prescribed at ultrahigh doses such as to achieve adequate organ protection [46,47] and because of high tolerability [20], the maximum achievable blood pressure reduction with a given substance might be a better basis for an efficacy comparison. There are however no randomized controlled studies to address this issue and indirect comparisons from multiple trials are always limited by differences in baseline blood pressure and patient characteristics.

**Table 8:** Recommended dose, defined daily dose (DDD) and doses with equivalent efficacy of angiotensin receptor blockers based on diastolic blood pressure reduction [42]

	**Recommended daily dose as to SPC**	**Defined Daily Dose (WHO)**	**Equi-effective dose **[41]
Candesartan	8	8	16
Eprosartan	600	600	800
Irbesartan	150	150	150
Losartan	50	50	100
Olmesartan	10	20	20
Telmisartan	40	40	40
Valsartan	80	80	160

### Value of fixed dose combinations?

The majority of patients with hypertension receive combination antihypertensive therapy. Current guidelines of the ESH/ESC recommend combination treatment for patients uncontrolled on monotherapy or in patients with blood pressure values on presentation exceeding 20/10 above the respective patient target [6]. Fixed-dose combination of different drugs may help to increase patients' compliance and persistence in these cases. In hypertension, such combinations have the potential to improve disease control and avoid hypertension-associated morbidity, thus increasing effectiveness and lowering non-drug medical costs [48]. But most studies assessing fixed-dose combination did not assess patients' compliance and persistence. However retrospective studies have shown that fixed-dose combination can lead to better compliance and persistence. This, in turn, leads to better health outcomes and fewer adverse medical events [49]. A recent study by Dickson and Plauschinat evaluated compliance with a treatment of the fixed-dose combination amlodipine/benazepril vs. free combination therapy in elderly Medicaid recipients [50]. The fixed combination treatment was associated with improved compliance and lower healthcare costs (defined as both drug and non drug costs) compared with a free combination.

## Conclusion

To evaluate drugs for the treatment of hypertension several key variables have to be considered. These variables include the blood pressure lowering effect, side effects, compliance and persistence with treatment, as well as drug costs and direct and indirect costs of medical healthcare. Given that the highest persistence with treatment results in the lowest non-drug costs, substantial cost savings and a reduction of morbidity might be expected making these drugs an attractive choice for long term treatment of hypertension.

## Competing interests

All authors have attended advisory boards and have held lectures for a number of pharmaceutical companies including sanofi-aventis and BMS.

## Authors' contributions

All authors have made substantial contributions to conception and design, or acquisition of data, or analysis and interpretation of data. PB has drafted the manuscript. JH revised the manuscript for important intellectual content. All authors have given final approval of the version to be published.
